# Effects of Chiral Polypeptides on Skyrmion Stability and Dynamics

**DOI:** 10.1021/acs.nanolett.4c05035

**Published:** 2024-12-16

**Authors:** Yael Kapon, Fabian Kammerbauer, Theo Balland, Shira Yochelis, Mathias Kläui, Yossi Paltiel

**Affiliations:** † Institute of Applied Physics, Faculty of Sciences, The Hebrew University of Jerusalem, Jerusalem 9190401, Israel; ‡ Institute of Physics, Johannes Gutenberg University Mainz, Staudingerweg 7, 55128 Mainz, Germany

**Keywords:** chiral-induced spin selectivity effect, spintronics, skyrmions, organic spintronics, magnetic memory

## Abstract

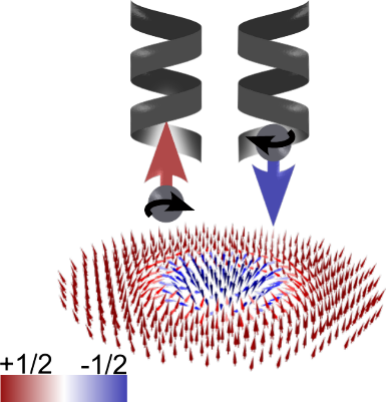

Magnetic skyrmions, topologically stabilized chiral spin textures in magnetic thin films, have garnered considerable interest due to their efficient manipulation and resulting potential as efficient nanoscale information carriers. One intriguing approach to address the challenge of tuning skyrmion properties involves using chiral molecules. Chiral molecules can locally manipulate magnetic properties by inducing magnetization through spin exchange interactions and by creating spin currents. Here, Magneto-Optical Kerr Effect (MOKE) microscopy is used to image the impact of chiral polypeptides on chiral magnetic structures. The chiral polypeptides shift the spin reorientation transition temperature, reduce thermal skyrmion motion, and alter the coercive field locally, enhancing skyrmion stability and thus enabling local control. These findings demonstrate the potential of chiral molecules to address challenges for skyrmion based devices, thus paving the way to applications such as the racetrack memory, reservoir computing and others.

Magnetic skyrmions, nanoscale, topologically stabilized chiral spin textures,^1,2^ have emerged as a promising avenue for next-generation information storage and processing technologies. Their unique properties, such as small size,^3^ high stability,^4−6^ and efficient current-driven motion,^7−10^ make them ideal candidates for applications in spintronics,^1,2,11,12^ where they can function as robust and energy-efficient data carriers. Recently, Skyrmions have been demonstrated to be promising candidates for new forms of energy-efficient computing,^13^ such as reservoir computing,^14−18^ and token-based Brownian computing.^19^ Thus, tuning skyrmion properties, such as the diffusion of skyrmions, for application in future spintronic devices is of increasing interest.

One intriguing approach to address the challenge of tuning skyrmion properties involves using chiral molecules. As illustrated in Figure 1, when a spin travels through a chiral structure that possesses a specific handedness, it can be polarized depending on the handedness^20−22^ (Figure 1A). Therefore, chiral molecules can interact with magnetic systems to induce spin-polarized currents^23−26^ and modify local magnetic properties through chiral-induced spin selectivity (CISS)^27−36^ effect. The metastable charge carrier polarization in chiral molecules induces spin polarization that interacts with the magnetic surface through exchange interactions.^37,38^ Given that CISS generates spin-polarized currents, one can expect that upon current injection, magnetic spin structures can be manipulated by the spin-polarized currents generated by the chiral molecules,^39^ offering a pathway to control skyrmion dynamics at the molecular level (Figure 1B). However, an open question is whether the molecules can affect spin structures such as skyrmions also without any externally injected charge currents.

**Figure 1:**
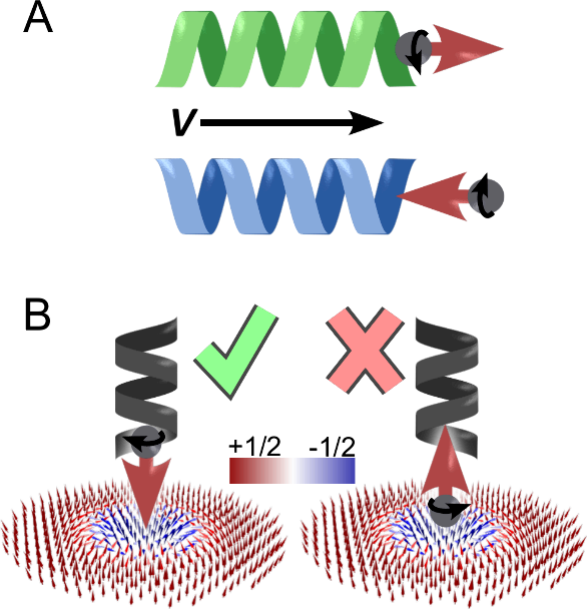
Chiral molecules and chiral magnetic structures. (A) When a spin (red arrows) travels through a chiral structure, the spin can be polarized according to the handedness of the molecule (left - green, right - blue). This interaction can locally alter the anisotropy of a magnetic system, thereby modifying its magnetic properties. (B) Chiral magnetic structures can be manipulated by spin currents induced by chiral molecules (in the image - Néel-type skyrmion).

Consequently, in this study, we explore the impact of chiral polypeptides on magnetic skyrmions in an ultralow-pinning magnetic thin films. Using MOKE microscopy, we image the magnetic state, extract film properties, analyze skyrmion dynamics, and investigate the effects of chiral polypeptides on those properties. Our results demonstrate that the presence of chiral molecules can manipulate the stability and motion of skyrmions, shifting the spin reorientation transition (SRT) temperature, attenuating thermal motion, and altering the coercive field locally. These findings highlight the potential of chiral molecules to overcome current obstacles in skyrmionics, paving the way for advanced magnetic devices such as racetrack memory^2,40,41^ and unconventional computing based on thermal diffusion.^15,16^

Ta(5)/Co_20_Fe_60_B_20_(0.9)/MgO(2)/Ta(2)/Au(5 nm) thin films with a perpendicular magnetic anisotropy (PMA) were prepared employing magnetron sputtering using a Singulus Rotaris sputter deposition tool. The films were designed with a very small anisotropy and to host skyrmions above room temperature big enough for optical imaging. The magnetization loops of the films without molecules and the corresponding domain imaging at different temperatures (25 °C – blue, 28 °C – orange, 36 °C – red) are presented in Figure 2A,B. The magnetization is normalized and proportional to the optical intensity. The films are ferromagnetic and perpendicularly magnetized at room temperature and have a spin reorientation transition (SRT) temperature above room temperature at around 33 °C. As the temperature increases, the domains’ size shrinks (Figure 2B left to middle) until above the SRT temperature skyrmions become the favored configuration (Figure 2B right). Skyrmions are identified in the MOKE image as isolated circular spin structures. The topologically nontrivial nature of these chiral spin structures was confirmed in similar films in previous studies.^10,42^ The SRT temperature can be detected by the temperature at which the perpendicular coercive field decreases to zero. A detailed description of the film preparation and sample-to-sample variability can be found in the Supporting Information (Figure S6).

**Figure 2:**
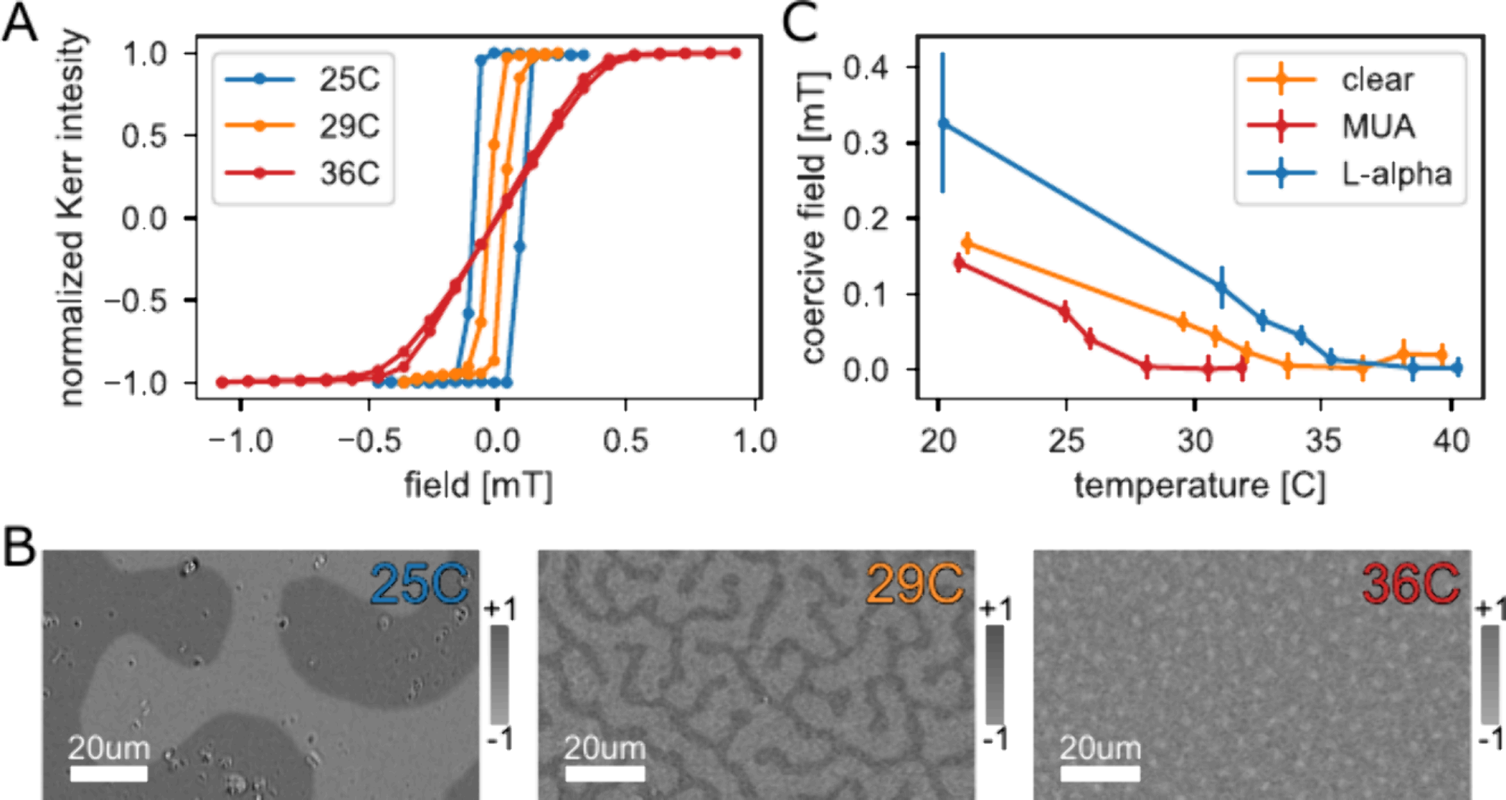
Shifted phase transition in the presence of chiral molecules. (A) Magnetization hysteresis loops measured for Ta/CoFeB/MgO/Ta/Au thin films at different temperatures (25 °C - blue, 29 °C - orange, 36 °C - red) using a MOKE microscope in the polar configuration. (B) Corresponding MOKE images at different temperatures. At room temperature, the sample is ferromagnetic with large magnetic domains. As the temperature increases, the coercive field decreases and domain size shrinks, eventually stabilizing magnetic skyrmions. The reduction of the coercive field to zero indicates the spin reorientation transition (SRT) temperature. (C) Coercive field as a function of temperature for a clean sample (orange), a sample with adsorbed achiral molecules (red), and a sample with adsorbed chiral molecules (blue). The SRT temperature increased by 3 °C with chiral molecules and decreased by 5 °C with achiral molecules, indicating induced changes in the perpendicular magnetic anisotropy. The error bars are standard error of mean, calculated from multiple measurements of the same sample.

To observe the effects of chiral polypeptides on the magnetic properties of the film, L-α helix polyalanine (L-AHPA) was adsorbed on the surface of the film to create a monolayer. As a reference, a sample with a-chiral 11-mercapto undecanoic acid (MUA) monolayer and a sample that underwent the same cleaning procedure without the molecule deposition were prepared. Multiple magnetization loops were taken for each sample and the coercive field of each magnetization loop was found by fitting a step function to each side of the loop (see Figures S3 and S4 in Supporting Information S2 for more information). The average coercive field of each sample at different temperatures is presented in Figure 2C (L-AHPA – blue, clear–orange, MUA – red).

The a-chiral MUA reference is used to isolate the effects of chirality from any chemical modification due to the adsorption process. It connects to the Au layer via a thiol Au covalent bond providing a similar adsorption mechanism and molecular length to the AHPA. Adsorption of MUA molecules reduce the coercive field of the sample and the film reaches SRT at a lower temperature. In contrast, at room temperature, the coercive field of the film with adsorbed chiral molecules is twice as large as the film without the molecules. In both films, with and without molecules, the coercive field decreases until it reaches zero in the SRT. Throughout the transition, the film with the chiral molecules has a higher coercive field and it reaches zero at higher temperature. The SRT temperature of the sample without molecules is found to be 33 °C while the sample with the L-AHPA monolayer has a higher SRT temperature of 38 °C.

To understand the effect of the chiral polypeptides on the skyrmion dynamics, we investigate the thermal motion of skyrmions in specifically engineered low-pinning Ta(4)/Co_20_Fe_60_B_20_(0.9)/Ta(0.08)/MgO(1.5)/Ta(2)/Au(3 nm) stacks. Chiral polypeptides were adsorbed on marked areas on the sample using electron beam lithography. Using selective adsorption, we were able to image in one image both areas with molecules and without (see Supporting Information S6 for more information). This was done to mitigate any issues arising from sample-to-sample variability making our results more robust. Using MOKE microscopy, we observed the behavior of skyrmions in real-time under varying out-of-plane magnetic fields to tailor their density and radius. An example frame from the video is displayed in Figure 3A, with skyrmion positions marked in blue for the areas with the chiral molecules and orange without. Our study compares skyrmion thermal dynamics in areas with adsorbed chiral molecules to areas without the molecules on the same film and under identical conditions. The experimental setup and methodology details are provided in the Methods section.

**Figure 3:**
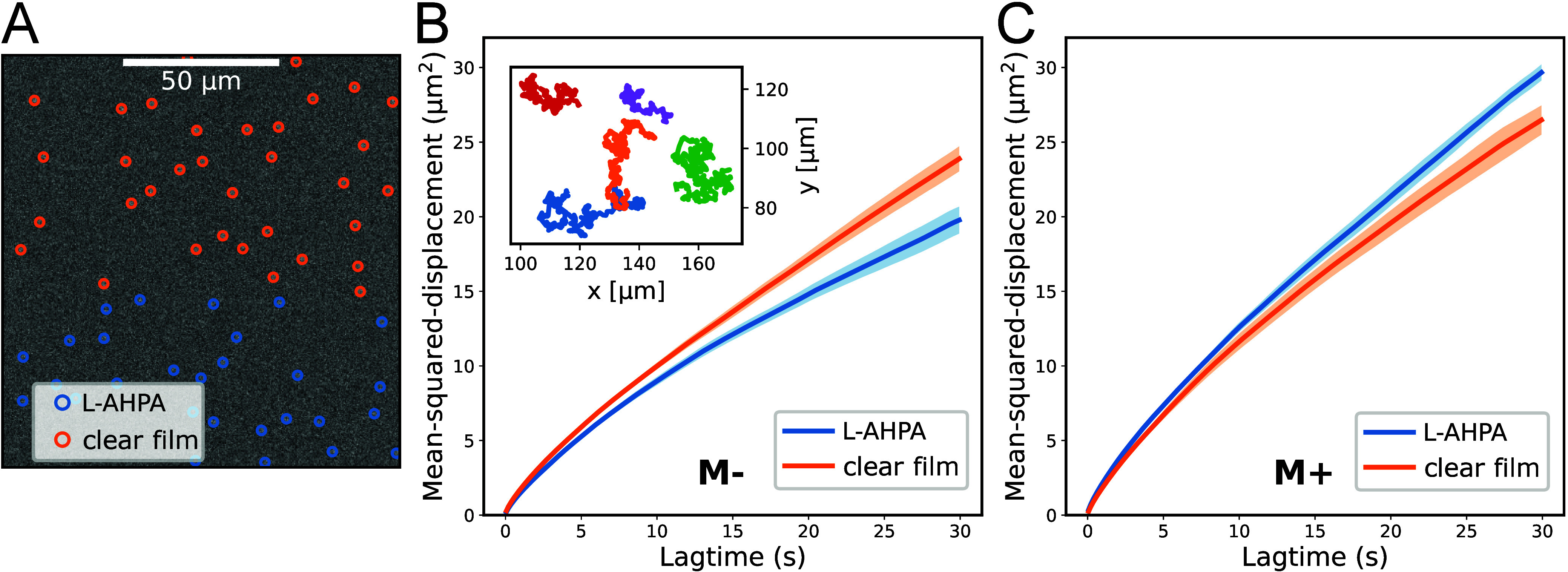
Skyrmion diffusion in the presence of chiral molecules. A video of skyrmion motion was taken, tracking the skyrmion positions in areas of the film with adsorbed molecules (blue) and without molecules (orange). (A) An example frame from the video, with the skyrmion position marked in blue. (B) The mean square displacement (MSD) of the skyrmions in an area with (blue) and without (orange) adsorbed chiral molecules for a negative applied field. The shaded areas display the standard error of the mean. Inset - examples of the skyrmion trajectories. (C) MSD for areas with (blue) and without (orange) adsorbed chiral molecules for positive applied field.

Skyrmions were monitored under constant conditions, exhibiting random motion throughout the sample. To quantify their diffusive motion, we tracked individual skyrmions and analyzed their trajectories. An example of typical random skyrmion trajectories is illustrated in the inset of Figure 3B and a video of the skyrmion motion is presented in the Supporting Information (Supplementary Video 1). The mean square displacement (MSD) of the skyrmions was calculated, assuming their motion could be modeled as pure diffusion—rigid particles with no correlations—similar to previous works^13,15,16^ The MSD is then simply given by MSD = ⟨|***R***(*t*)-***R***(0)|^2^⟩ The average MSD, proportional to time for pure diffusion, was plotted to further understand the skyrmion dynamics.

The MSD of skyrmions in regions with adsorbed chiral molecules (L-AHPA), shown in blue and without molecules (orange) for a negative applied magnetic field is plotted in Figure 3B. The results demonstrate that areas with adsorbed molecules exhibit reduced thermal motion, indicating that these molecules act as pinning sites for the skyrmions, thus reducing their mobility. However, an increased diffusion is observed when the experiment is performed while applying a positive magnetic field, as seen in Figure 3C. Furthermore, the MSD in the region of no adsorbed molecules, i.e. the clear film, is the same between the two sets of measurements, indicating a crucial role of the molecule and the field polarity. A sample with a-chiral MUA was studied as well and no difference in diffusion was measured with and without the molecules indicating the effect is due to the chirality (see Supporting Information S5 for the results). We note that any possible magnetic field gradient, induced by a nonuniform magnetic field, does not explain the observed behavior. As the qualitative difference in diffusion should be the same whether the field is positive or negative, a detailed description can be found in the Supporting Information.

Finally, we explore the local effects of chiral polypeptides by patterning a track in Ta(5)/ Co_20_Fe_60_B_20_(0.9)/MgO(2)/Ta(2)/Au(5 nm) film and selectively adsorbing the chiral molecules on different areas on the track and simultaneously measuring adjacent areas in the film with and without molecules (see inset in Figure 4). Magnetic hysteresis loops were taken for the thin films patterned with a monolayer of L molecules (blue) or D molecules (green), alongside the areas near the monolayer, without molecules (orange). The magnetization loops are shown in Figure 4A (L-chirality) and 4B (D-chirality). Magnetization loops were simultaneously taken from the same image in different areas, with and without the molecules, to ensure consistent conditions. The area with L molecules exhibits a wider loop i.e. a higher coercive field compared to the adjacent area without molecules. It is important to note the symmetric change of the coercive fields of the hysteresis loop, in contrast to the previously observed asymmetric shift in the hysteresis.^30^ A sample with the opposite chirality of molecules (D) was studied as well to understand how the opposite induced spin affects the sample. The D-chirality is also meant to isolate the effects of chirality from any chemical modification due to the adsorption process. The area with D molecules has a narrower loop, i.e. lower coercive field, than the adjacent area without molecules. These findings suggest that the chiral polypeptides change the PMA depending on the chirality of the molecules.

**Figure 4:**
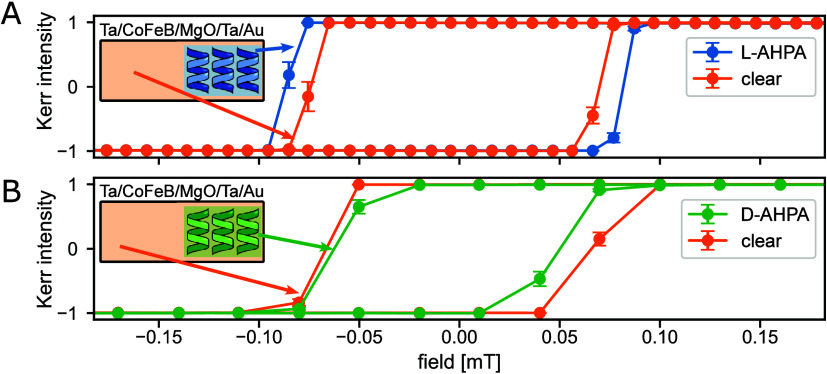
Local changes in coercive field. Magnetization loops at *T* = 30.8C (A), 31.9C (B) of a Ta/CoFeB/MgO/Ta/Au thin film patterned with a monolayer of (A) L molecules (blue) or (B) D molecules (green) in the monolayer area compared to the clear area near the monolayer (orange). Magnetization loops were taken from the same image on different areas simultaneously as illustrated in the inset. The area with L molecules has a wide magnetization loop while the area with D molecules has a narrower loop compared to the adjacent area without molecules. The error bars are standard error of mean, calculated from multiple measurements of the same sample.

A Ta(4)/Co_20_Fe_60_B_20_(0.9)/Ta(0.08)/MgO(1.5)/Ta(2)/Au(3 nm) film was patterned with chiral polypeptides to achieve visible changes between the areas with and without the patterned molecules. This film has stripe domains at room temperature and its magnetic properties change slower with temperature as opposed to the previous film. This allows us to directly image the effect of the molecules on the film. The number of skyrmions/stripe domains was investigated at different temperatures and fields close to magnetic saturation. A MOKE image of two such conditions (top −24.7C; 0.23 mT, bottom −24.7C; −0.1 mT) is presented in Figure 5A, where blue marks the area with adsorbed L molecules and orange the area without molecules. The contrast difference is due to different background images used. The area with adsorbed chiral molecules contains fewer skyrmions (encircled in red) and domains than the adjacent area without molecules, supporting the notion that the adsorbed molecules dynamically influence skyrmion density and distribution. In Figure 5B we present the number of isolated domains (such as skyrmions and small stripe domains) at 20.25 mT magnetic field as a function of temperature (24.7C, 27.8C. 29.3C) for areas with (blue) and without (orange) adsorbed molecules. Here, the term “isolated domains” refers to domains that are not yet magnetically flipped—i.e., a higher magnetic field is required to reach saturation. As the temperature increases, a higher magnetic field is needed to saturate the film thus isolated domains remain stable at higher fields. The adsorbed molecules cause a shift in this trend generating fewer locally isolated domains. As shown in Figure 5C, this trend persists at different fields as well. The number of domains as a function of the magnetic field at different temperatures (24.7C – blue, 27.8C – green, 29.3C – pink) in areas with (solid line) and without (dashed line) adsorbed molecules is presented in Figure 5C.

**Figure 5:**
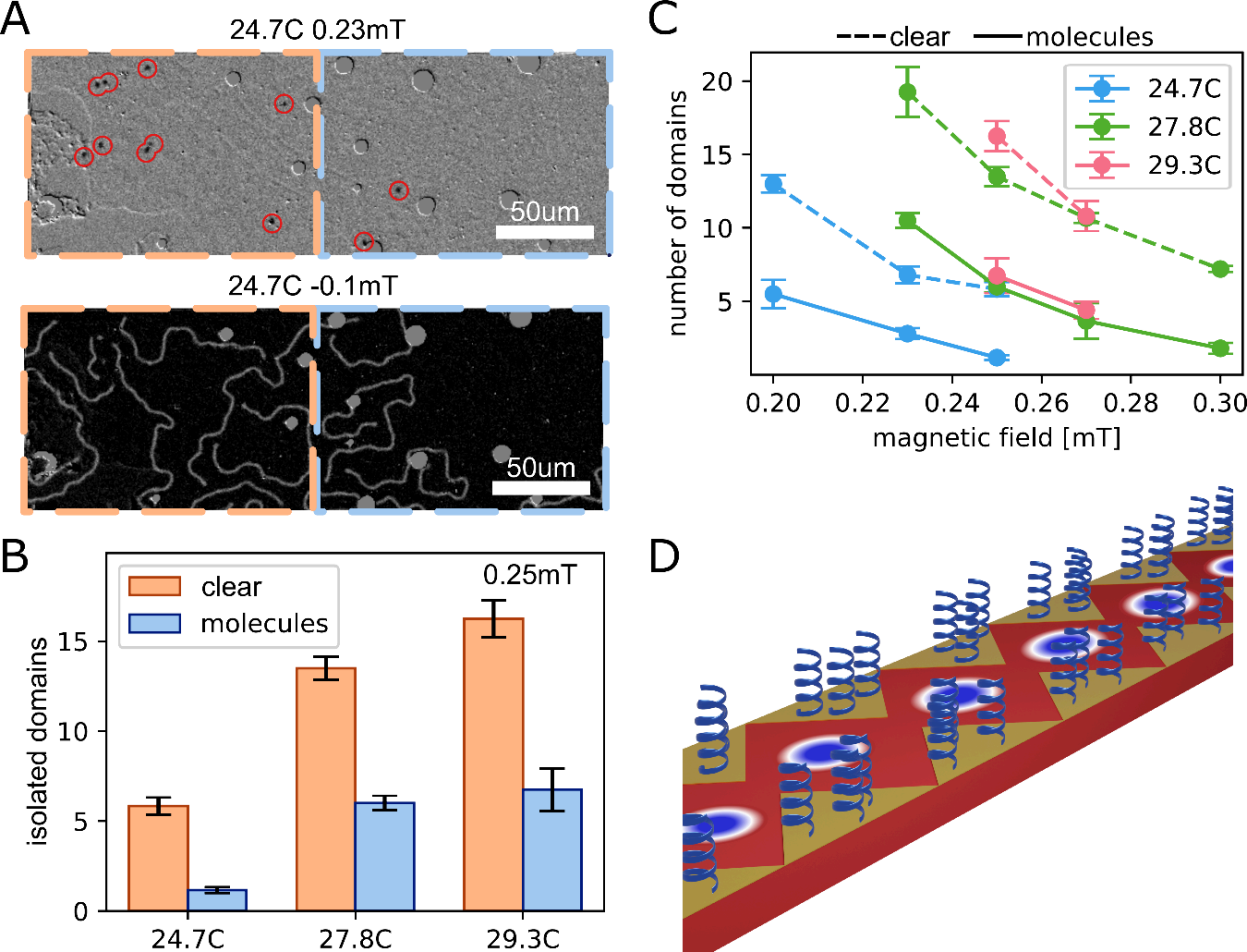
Patterning skyrmion tracks. (A) MOKE image of Ta/CoFeB/Ta/MgO/Ta/Au thin film with patterned adsorbed molecules on the right side (orange - clear film and blue - with adsorbed L molecules) at two different conditions: top −24.7C; 0.23 mT, bottom −24.7C; −0.1 mT. (B) number of isolated domains at 20.25 mT magnetic field at different temperatures (24.7C, 27.8C. 29.3C) for areas with (blue) and without (orange) adsorbed molecules. (C) number of isolated domains at different magnetic fields and temperatures (24.7C – blue, 27.8C – green, 29.3C – pink) for areas with (solid line) and without (dashed line) adsorbed molecules. (D) Proposed use of chiral molecules in a racetrack memory device. The molecules can be adsorbed selectively in notches at the sides of the device controlling the motion of skyrmions in the nanotrack. The error bars are standard error of mean.

In our study, we find that chiral polypeptides significantly impact the magnetic properties of magnetic thin films. This is evident from the shifted spin reorientation transition temperature and the effects on the thermal motion of skyrmions. We find consistent behavior throughout various types of measurements and scales, demonstrating a robust interaction between the chiral molecules and the magnetic properties of the material. A summary table of all samples and results is presented in the Supporting Information (Supporting Information S8). Especially, the enhancement or suppression of thermal skyrmion motion depends on the magnetic field direction, and therefore the skyrmion polarity shows a chirality dependence. This is in agreement with the previously measured asymmetric exchange between the chiral molecules and PMA magnetic thin films.^30^ A possible explanation is the small locally induced magnetic moments at the interface of the molecule and Au layer as previously observed.^43−45^ These moments would generate a constant effective field in the areas with molecules affecting the skyrmion size and thereby the skyrmion diffusion. The necessary effective field can be approximated by comparison with previous studies.^13,46^ A 10% change in MSD could be facilitated by a field change as small as 2 μT, which is within the error of the applied magnetic field. This takes into account the sample-specific change in skyrmion radius with magnetic field.

The symmetric increase in the coercive field is unexpected, as a uniform layer of magnetic moments typically results in an asymmetric magnetic response. Indeed, Ben Dor et al. observed a horizontal shift in the hysteresis loop. It is important to note the difference in magnetic samples: while the study by Ben Dor et al. focused on ordered out-of-plane domains with large PMA, this study involves a soft magnetic system with low PMA and an additional MgO layer that separates the magnetic layer from the molecules electrically. Such a layer prevents direct electronic coupling possibly impacting and reducing the effective fields generated. We note that the stack used here is close to the transition to an in-plane anisotropy, capable of hosting complex spin textures, such as skyrmions that are most sensitive to small variations in the magnetic properties. Furthermore, a similar symmetric increase in the coercive field was also observed in Ni with an in-plane anisotropy,^34^ suggesting that the orientation of magnetic moments can influence the coupling between the molecules and the magnetic film.

In our study, a vertically stacked magnetic film where the DMI vector is parallel to the film plane was used. While the molecules have some tilt angle and so they have some in-plane effect, it will be interesting to explore the connection between the direction of the Dzyaloshinskii-Moriya (DMI) vector and the molecular axis of the molecules, similar to previous studies exploring the connection between the molecules’ molecular axis and the ferromagnet’s easy axis.^47^ In addition, the molecules’ induce a surface effect, so working with low-dimensional materials hosting skyrmions,^48^ in which the interface can be tunable, can lead to understanding the nature of the interaction better.

A potential application of chiral molecules in racetrack memory devices is proposed in Figure 5D. By selectively adsorbing these molecules in notches on the sides of the device, it is possible to control the motion of skyrmions within the nanotrack, similarly to theoretically proposed devices.^49^ This selective patterning could pave the way for more precise skyrmion manipulation, enhancing the performance and reliability of skyrmion-based memory and logic devices.

The mechanisms behind the magnetization changes induced by chiral polypeptides can be attributed to several factors. Previous studies have shown that the adsorption of chiral polypeptides can locally change the perpendicular magnetic anisotropy (PMA) in the material.^29,34,50^ Modifications of the PMA have been shown to effectively manipulate skyrmions generation and annihilation, and control their movement.^51^ Additionally, a possible alteration of the DMI has been suggested.^52,53^ DMI changes due to chemical adsorption have been shown for various elements.^54,55^ Here the molecules are bigger and the effects of the adsorption of chiral molecules remain speculative and require further experimental work. Future work will need to focus on direct measurements of the chiral interactions to fully elucidate the underlying mechanisms and harness the full potential of chiral molecules in skyrmionics. This understanding could lead to an enhancement of the interaction of chiral molecules and magnetic thin films.

In summary, we have demonstrated that the adsorption of chiral polypeptides can manipulate chiral magnetic structures such as domains and skyrmion in magnetic thin films. Using MOKE microscopy, we observe that the adsorbed chiral molecules reduce or increase skyrmion diffusion depending on the magnetic field direction and locally change the magnetic properties of the film such as shifted spin reorientation transition temperature, attenuated thermal motion of skyrmions, and effectively reducing the density of skyrmions and domains in the material. These effects suggest an interaction between the chiral polypeptides and skyrmions, paving the way for using chiral polypeptides and materials in skyrmion-based memory and logic devices.

## Supporting Information Available

Details of the sample preparation, MOKE measurements, detection of skyrmion motion, and additional figures and tables (PDF)
video of the skyrmion diffusion (MP4)
