# Supplementary material for: Effects of Chiral Polypeptides on Skyrmion Stability and Dynamics
Source: Nano Lett. 2024 Dec 16;25(1):306–12. doi: 10.1021/acs.nanolett.4c05035 (PMC11719627; doi:10.1021/acs.nanolett.4c05035)
Supplement: Supplementary file 1 — nl4c05035_si_001.pdf [file nl4c05035_si_001.pdf]

Effects of Chiral Polypeptides on Skyrmion Stability and Dynamics

Yael Kapon[1,+], Fabian Kammerbauer[2,+], Theo Balland[2], Shira Yochelis[1], Mathias Kläui[2,*], Yossi Paltiel[1,*]

[1] Institute of Applied Physics, Faculty of Sciences, The Hebrew University of Jerusalem, Jerusalem 9190401, Israel

[2] Institute of Physics, Johannes Gutenberg University Mainz, Staudingerweg 7, 55128 Mainz, Germany

[+]Contributed equally

[*]Corresponding authors

Mathias Kläui klaeui@uni-mainz.de

Yossi Paltiel paltiel@mail.huji.ac.il

Supporting Information

S1 Experimental procedures

Sample preparation

The Ta(5)/ Co20Fe60B20 (0.9)/MgO(2)/Ta(2)/Au(5nm) and Ta(4)/Co20Fe60B20(0.9)/Ta(0.08)/MgO(1.5)/Ta(2)/Au(3nm) thin films were deposited by magnetron sputtering using a Singulus Rotaris sputtering system on thermally oxidized silicon substrates. The base pressure in the chamber is around 10^{-8} mbar. Films were deposited in an Argon atmosphere of around 10^{-3} mbar. The film thickness is estimated from sputter rates determined by X-ray reflectivity measurements.

The film to measure local changes in the coercive field was patterned using ion beam milling (AJA, USA | ATC Series). 70nm Ti hard mask (created by photolithography and evaporation) was created followed by etching of the sputtered layers using ion milling at 10 degrees with a power setting of 600 watts, for 15 minutes at 60-second intervals until the SiO layer of the substrate was exposed. The leftover mask was removed by HF.

For the skyrmion dynamics sample Ti(10nm)/Au(50nm) markers were evaporated for convenient orientation in the film.

36 - L/D α-helix polyalanine (L/D-AHPA) [[H]-C[AAAAK]$_7$-[OH]] molecules (C stands for cysteine, A for alanine, and K for lysine), as well as a-chiral 11-mercapto undecanoic acid molecules were manufactured by Sigma–Aldrich. A 1mM solution was prepared in ethanol and used in the experiments.

Sample cleaning was performed with boiling acetone for 10 minutes followed by boiling isopropanol for 10 minutes and subsequently water. The samples were dried by a Nitrogen gun and then put on a hot plate at 80ºC to evaporate any residual solutions.

To prepare the exposed Au surface for adsorption, it was soaked in ethanol for 20 minutes to reduce the produced oxides. Afterwards, the L/D α-helix polyalanine (L/D-AHPA), or a-chiral 11-mercapto undecanoic acid molecules were chemically adsorbed through their tiol edge group onto the surface via an overnight soak in 1 mM solution of the molecules in ethanol in a nitrogen environment[30]. Residue molecules were removed by washing in ethanol. Monolayer characterization by XPS (table S2&3) and AFM (Fig.S5) is detailed in supplementary information S3

Selective adsorption of L/D α-helix polyalanine (L/D-AHPA), or a-chiral 11-mercapto undecanoic was obtained by defining square structures into a poly(methyl methacrylate) (PMMA) resist layer using e-beam lithography, where the surface was exposed. Monolayer adsorption was done as described above. The PMMA was removed by washing the sample with acetone to dissolve the PMMA, then washed in ethanol again and dried in a Nitrogen environment.

MOKE imaging and magnetometry

MOKE imaging and magnetometry were performed by a commercial Evico Magnetics GmbH magnetooptical Kerr microscope. The measurements were taken in the polar configuration. An out-of-plane magnetic field was generated by electromagnets obtained from the microscope supplier powered by a Kepco BOP 100-4DL power supply. For the optical imaging of magnetic samples, 20X or 50X commercial Zeiss objective lenses were used. For the MOKE imaging, first, a magnetic field was applied to saturate the sample. Then, the saturated image was subtracted from the optical image to enhance the magnetic image contrast. Mechanical vibrations of the sample were actively stabilized by a piezo stage. For the Kerr hysteresis measurements, the optical intensity was averaged over a chosen area and then normalized to saturation magnetization. The temperature was controlled by a Peltier element and measured by a Pt Resistance Temperature Detector in a temperature-stabilized flow box environment.

Detection of skyrmion motion

We use a developed machine-learning-based detection of skyrmions to detect the skyrmions frame by frame[53]. The resulting skyrmion positions have been linked to trajectories by employing the trackpy package[54] The mean-squared-displacement (MSD) is calculated from the trajectories by first removing any trajectories below 600 frames in length and removing pinned particles whose standard deviation in x or y coordinates is below 5 px. The full image area is divided into two sections, one part with and one part without molecules. The MSD is then calculated for trajectories in each of the areas separately, hereby, a sliding window is used to calculate lag times up to 30s $\mathrm{MSD} = \sum_{k=0}^{\mathrm{t=30s}} \langle |\boldsymbol{R}(t) - \boldsymbol{R}(0)|^2 \rangle$

S2 Magnetic field gradient effects in the Skyrmion diffusion

The skyrmion diffusion is analyzed in samples with selective adsorption of molecules. While this gives the unique opportunity to measure the areas with and without molecules on the same film that have experienced the same treatment, not all parameters are the same.

As shown in Figure 3A of the main manuscript, the videos of skyrmion diffusion have been taken with molecules on the bottom side of the field of view and the area without molecules on the bottom side of the field of view. Owing to this geometric configuration, horizontally separated regions may be subjected to vertical field gradients, potentially affecting skyrmion diffusion—a property that is highly sensitive due to its exponential dependence on the energy landscape.

We, therefore, performed control experiments where we took videos of the adsorbed area with molecules once at the bottom and once on top, see Figure S1A. A magnetic field gradient in the horizontal direction would not affect the data as areas with and without molecules are affected in the same way.

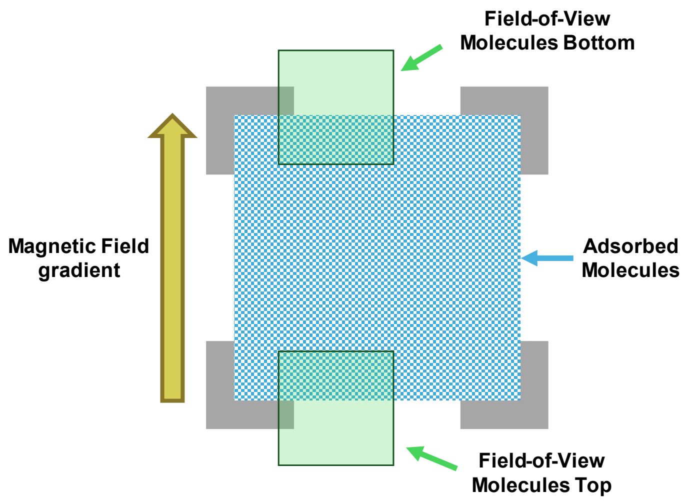

Fig. S1: Sketch of the chosen field of views in the vertical direction (not to scale). The dashed area represents the area of selective adsorption of molecules, which is marked with 100 nm thick Au markers at the corners.

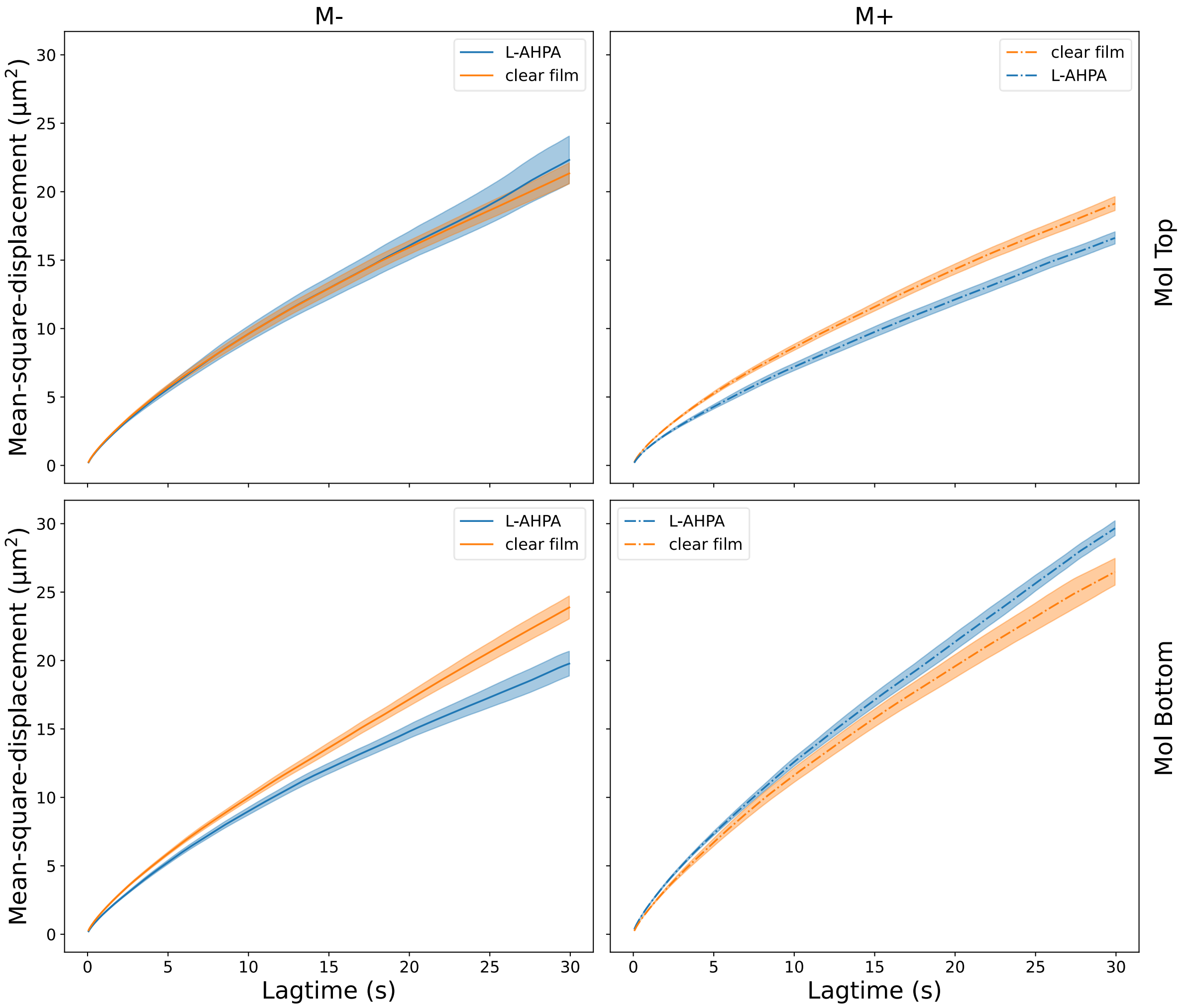

Fig S2: MSD Grid plot of the 4 different measurement configurations. The two top plots are additional measurements taken using another field of view as specified in Figure S1. The colored areas represent the standard error of the mean.

In Figure S2 we display the mean-squared-displacement (MSD) for the two fields of views showcased in S1 and the two magnetic field directions for the areas with L-AHPA and the clear film, respectively. The exact field values and temperatures of the measurements can be found in Table S1. We see that there is a difference in MSD between two respective measurements, i.e. M+ and M-, in the same field of view. We want to focus on the clear film first, as this functions as a reference point. For the clear film case, the diffusion is comparable between M+ and M- which means the skyrmion size for M+ and M- are similar, as diffusion is mainly governed by skyrmion size and temperature [citation]. However, the areas with adsorption molecules do not follow this pattern. The temperatures of the two measurement sets are the same, and the clear film areas are comparable between the two videos excluding the temperature as origin. A magnetic field gradient also cannot explain this behavior within the same field of view but in different magnetic field directions. The reason is, that the size of the skyrmions is determined by the absolute magnetic field. The magnetic field gradient, in terms of absolute field, is in the same direction for M+ and M-. Therefore, if a gradient is the source of the difference in diffusion, it should be the same for both field directions. Which is not observed for either of the two field-of-views. We, therefore, exclude a field gradient as a possible origin of the difference in skyrmion diffusion.

Configuration	Field-of-view	Field direction	Temperature (K)	Magnetic field (mT)
1	Mol Top	M-	297.8	-0.192
2	Mol Top	M+	298.2	0.69
3	Mol Bot	M-	298.6	-0.181
4	Mol Bot	M+	298.6	0.66

Table S1: Configuration of measurements. With the field-of-views as displayed in Figure S1. For each configuration, 5 videos of 5 minutes in length were taken.

S3 analysis of MOKE hysteresis loops and coercive field

Multiple hysteresis loops were taken by averaging the intensity over a given area. Each curve was normalized and corrected for drifts and Faraday's rotation. The averaged loops of areas with and without molecules at different temperatures are shown in Fig. S3.

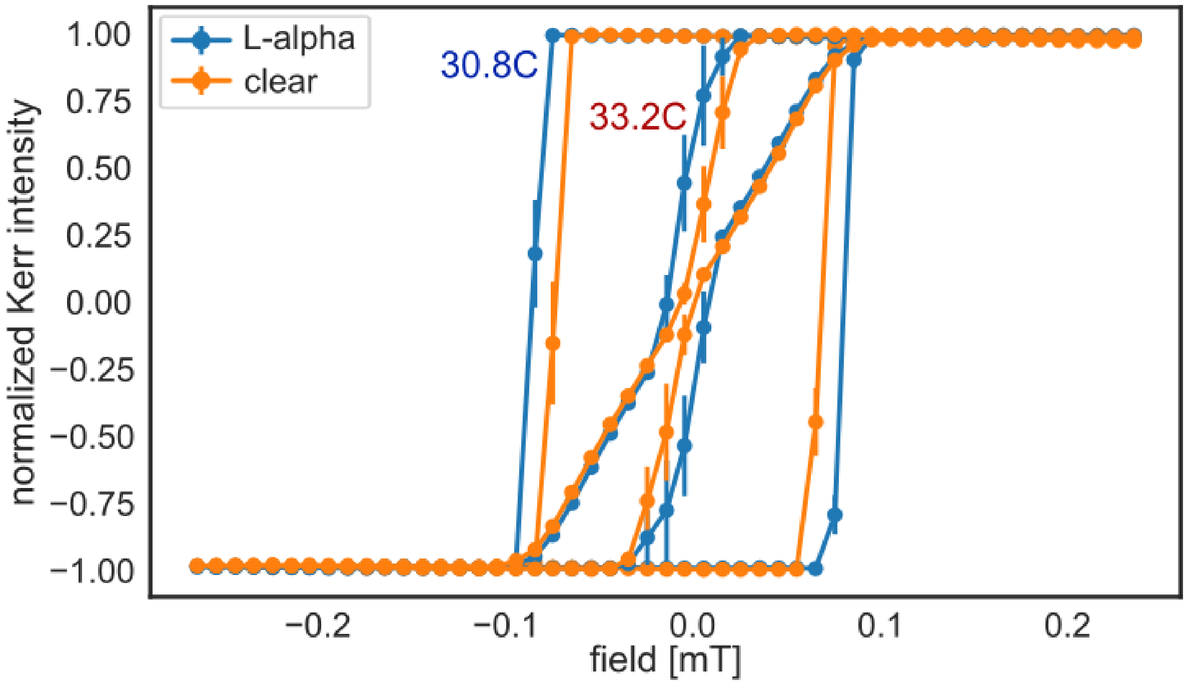

Fig. S3: Averaged Intensity vs. magnetic field hysteresis loops taken from different temperatures. The clear sample is marked in orange, while the area with the L-alpha helix polyalanine monolayer is marked in blue.

Each hysteresis loop was divided into forward (field sweep from - to +) and backward (field sweep from + to -) parts and a sigmoid function was fitted to each side:

$$I = M_s \tanh\left(\frac{H - H_c}{\alpha}\right)$$

The coercive field for each loop was calculated as $H_c = \frac{H_c^f - H_c^b}{2}$

The coercive field at a given temperature was the average coercive field of different loops with the error taken as the standard error of the mean.

Examples of the fitting curves are presented in Fig.S4.

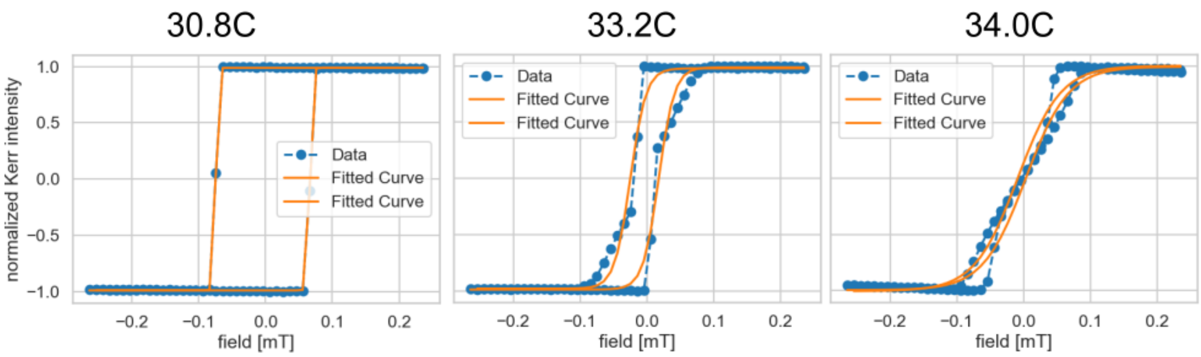

Fig. S4: Fitting examples for finding the coercive field for temperatures of 30.8°C, 33.2°C and 34°C. hysteresis loop is marked in blue while the forward and backward fitting is marked with orange.

S4 monolayer characterization

Monolayer characterization was performed by X-ray photoelectron spectroscopy (XPS) and Atomic Force Microscopy (AFM).

Table S2: Quantification of X-ray Photoelectron Spectroscopy (XPS) Results for Elemental Composition in Alpha-Helix Polyalanine Monolayer on Ta(5)/ Co20Fe60B20 (0.9)/MgO(2)/Ta(2)/Au(5nm)

Sample: **alpha-helix polyalanine monolayer on Ta(5)/ Co20Fe60B20 (0.9)/MgO(2)/Ta(2)/Au(5nm):**

	Atomic conc. [%]	Error [%]	Mass conc. [%]	Error [%]
Au 4f	35.51	0.47	89.11	0.21
S 2p	1.63	0.23	0.67	0.09
C 1s	48.81	0.66	7.47	0.14
N 1s	4.03	0.70	0.72	0.13
O 1s	10.01	0.46	2.04	0.10

Sample: **Ta(5)/ Co20Fe60B20 (0.9)/MgO(2)/Ta(2)/Au(5nm) without molecules**

	Atomic conc. [%]	Error [%]	Mass conc. [%]	Error [%]
Au 4f	46.15	0.41	92.60	0.12
S 2p	2.00	0.20	0.65	0.07
C 1s	41.70	0.46	5.10	0.08
N 1s	0.31	0.26	0.04	0.04
O 1s	9.84	0.40	1.60	0.07

The increased concentrations of O, C, and especially N in the alpha-helix polyalanine monolayer sample, in contrast to the clean substrate, signify the appearance of polyalanine molecules.

Table S3: Quantification of X-ray Photoelectron Spectroscopy (XPS) Results for Elemental Composition in Alpha-Helix Polyalanine Monolayer on Ta(4)/Co20Fe60B20(0.9)/Ta(0.08)/MgO(1.5)/Ta(2)/Au(3nm)

Sample: **alpha-helix polyalanine monolayer on Ta(4)/Co20Fe60B20(0.9)/Ta(0.08)/MgO(1.5)/Ta(2)/Au(3nm):**

	Atomic conc. [%]	Error [%]	Mass conc. [%]	Error [%]
Au 4f	15.04	0.19	72.53	0.29
S 2p	0.62	0.11	0.49	0.09
C 1s	57.46	0.57	16.90	0.28
N 1s	9.33	0.31	3.20	0.11
O 1s	17.56	0.40	6.88	0.15

Sample: **Ta(4)/Co20Fe60B20(0.9)/Ta(0.08)/MgO(1.5)/Ta(2)/Au(3nm) without molecules**:

	Atomic conc. [%]	Error [%]	Mass conc. [%]	Error [%]
Au 4f	31.20	0.39	86.77	0.21
S 2p	1.24	0.18	0.56	0.08
C 1s	44.53	0.66	7.55	0.17
N 1s	3.17	0.53	0.63	0.11
O 1s	19.86	0.34	4.49	0.07

The increased concentrations of C and especially N in the alpha-helix polyalanine monolayer sample, in contrast to the clean substrate, signify the appearance of polyalanine molecules.

AFM topography scan in tapping mode was used as another verification of the adsorption. Selectively adsorbed 5*5um squares of alpha-helix polyalanine were prepared using e-beam lithography as described in the methods section. The topography scan showed consecutive 5*5um elevated squares (the monolayer's elevated topography) indicating a successful adsorption.

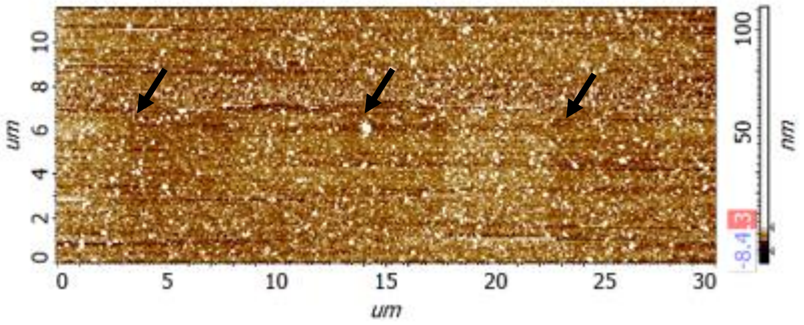

Fig. S5: AFM topography of Ta(5)/ Co20Fe60B20 (0.9)/MgO(2)/Ta(2)/Au(5nm) sample showing elevated 5*5um squares (the monolayer). the black arrows point to the top right corner of each square.

S5 Skyrmion diffusion with selective adsorption of achiral molecules

The observed change in skyrmion diffusion could be explained by a change in local anisotropy due to strain induced by the chemisorption of molecules. To ascertain whether this hypothesis is true and whether the effect is not related to the chiral property of the molecule, we perform the same experiment of skyrmion diffusion in samples with selective adsorption of achiral mercapto molecules. The analysis is performed in the same manner as for the chiral molecules. The resulting MSD plots are displayed in Figure S6. We see little difference between the two distinct areas, with molecules and without. Especially when the magnetic field is reversed the difference in MSD between mercapto and clear film is well within the error. Therefore, we assume a small to negligible effect from the adsorption of molecules alone. Compared to the adsorption of chiral molecules the changes are more pronounced further supporting the influence of the molecule chirality.

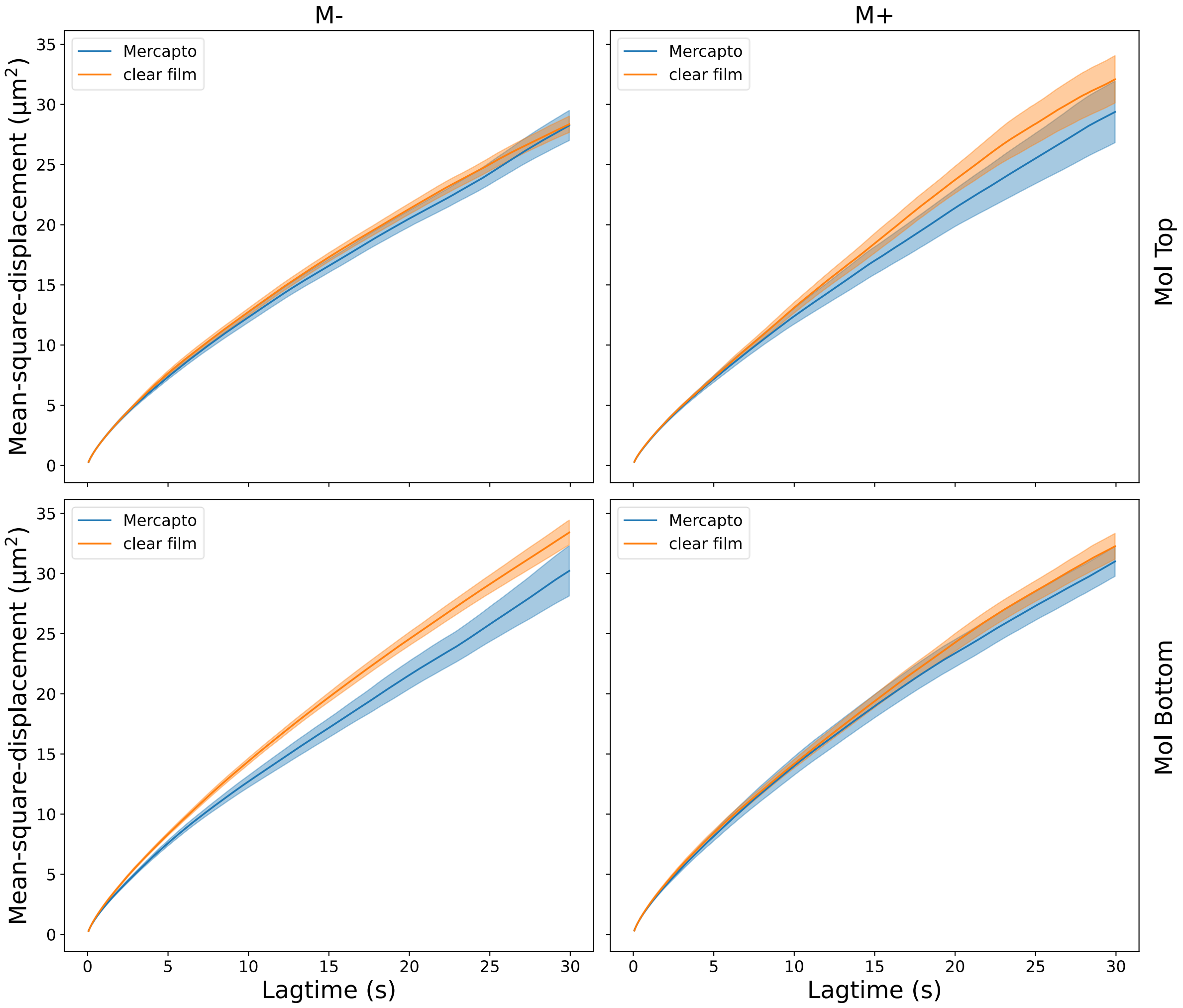

Fig S6: MSD grid plot of 4 different measurement configurations for achiral molecules. The fields of view, Mol Top and Mol Bottom, are specified in Figure S1. The colored areas represent the standard error of the mean.

S6 selective adsorption procedure

To further reduce sample-to-sample variability, data following Figure 2 were obtained from adjacent regions on the **same sample**, with and without molecule deposition by a method of adsorption on selective areas. Selective adsorption was achieved by spin-coating the samples with PMMA resist, opening regions via e-beam lithography, and adsorbing molecules onto these regions. The PMMA was then removed with acetone, leaving only the bonded molecules (as shown in Figure R4). This method allowed us to measure the area with and without molecules on the same sample simultaneously via MOKE microscopy. Figure R3F (from Figure 5A) shows an example of such a configuration, with molecules adsorbed on the right and a clear area on the left. We added this figure to the supporting information for clarification.

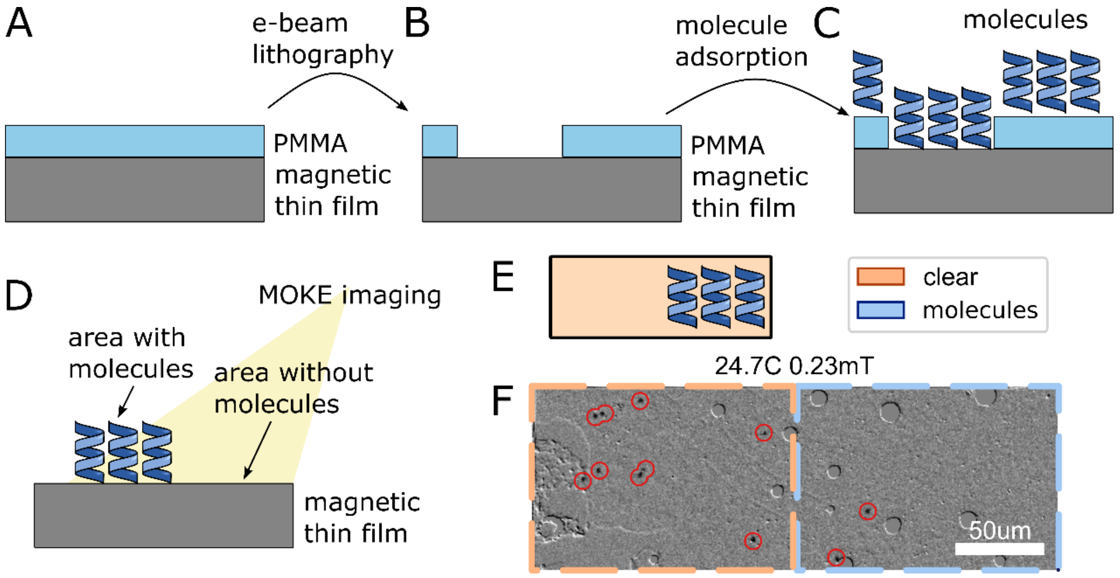

Figure S7: (A) The magnetic thin film samples were spin-coated with PMMA resist and (B) e-beam lithography was used to open selective areas in the PMMA before (C) the molecules were deposited onto the surface. The PMMA acted as a barrier between the molecules and the surface. (D) After adsorption, the PMMA was removed with acetone, washing the unbonded molecules as well and leaving the bonded molecules intact. (E) We are eventually left with a magnetic thin film with a specific area with adsorbed molecules which we can image and measure simultaneously with the area without the molecules using MOKE microscopy. (F) An example of MOKE image with the right area containing molecules adsorbed to the surface while the left area is without molecules is taken from Figure 5A.

S7 Sample-to-sample variability

For Figure 2 different samples have been measured which are taken from the same deposited wafer, nevertheless due to the sensitive nature of the film small variability still exists within these samples. We performed an additional measurement on a chip of about 35 mm x 35 mm size taken from the edge of this wafer. It therefore highlights the worst case of homogeneity of deposition, as homogeneity decreases with distance towards the center. We employ a laser MOKE setup to measure the chip in 5 mm steps in the x and y directions. The resulting hysteresis loop is displayed in Figure S8 below. We see a variation in the coercive field up to 0.05 mT, which is still smaller than the largest differences observed in Figure 2. This further strengthens the point of employing local adhesion of molecules, as in Figure 3,4 and 5. instead of sample-to-sample tests, as variations within the sample within 500µm, the field of view of the MOKE microscope setup, is too minor to be observable.

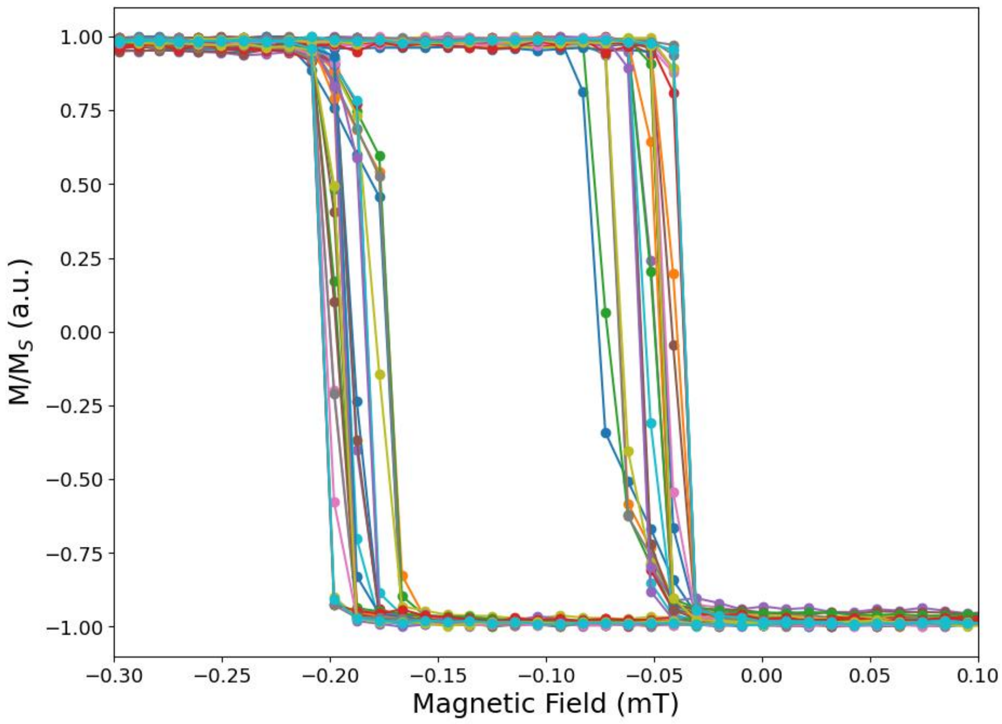

Figure S8: Hysteresis loops of a 35 mm x 35 mm chip extracted from the wafer containing the deposition of sample Ta(4)/Co20Fe60B20(0.9)/Ta(0.08)/MgO(1.5)/Ta(2)/Au(3nm) Each loop corresponds to a measurement taken within a 5 mm grid on the chip.

Sample-to-sample variability due to the fabrication and adsorption process was also checked. We present in Figure S9 magnetic hysteresis loops of the clear area of two samples after processing (the area without molecules in Figure 4). We find that the fabrication process did not create changes within the samples.

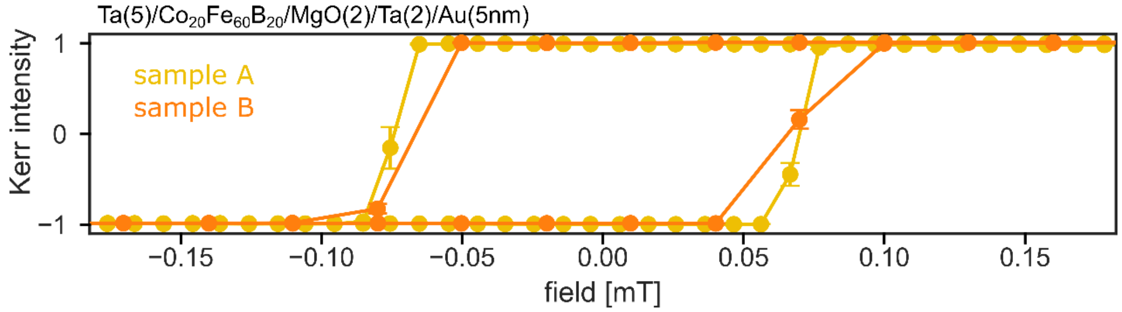

Figure S9 –Hysteresis loops from two different samples (A&B) that were used for Figure 4. Sample-to-sample variability post-fabrication and adsorption is small.

S8 Sample types and trends

In this study, we compare clear film properties (without molecules) to the film's properties after adsorption. A-chiral molecules and molecules of opposite chirality are used as references in different cases. A new set of samples was prepared for each measurement type. L-chiral molecules adsorption increases the coercive field locally, stabilizing the ferromagnetic phase. This effect happens both on a sample scale and locally after selective adsorption. The molecules change skyrmion dynamics

(increasing or decreasing motion depends on the applied field direction) and reduce skyrmion density. Table S4 is a summary table of all samples and measurements.

Table S4: summary table of all samples and measurements

**Molecule**	**Configuration**	**Observation**
Ta(5)/$Co_{20}Fe_{60}B_{20}$(0.9)/MgO(2)/Ta(2)/Au(5nm)		
Clear	Hysteresis	Baseline
L	Full Surface	Increased coercive field, higher SRT temperature
MUA	Full Surface	Decreased coercive field, lower SRT temperature
Ta(5)/$Co_{20}Fe_{60}B_{20}$(0.9)/MgO(2)/Ta(2)/Au(5nm)		
L	Selective Adsorption	Increased coercive field
D	Selective Adsorption	Decreased coercive field
Ta(4)/$Co_{20}Fe_{60}B_{20}$(0.9)/Ta(0.08)/MgO(1.5)/Ta(2)/Au(3nm).		
L	Selective Adsorption	Reduction in skyrmion diffusion at (-) field and increasing at (+) field
L	Selective Adsorption	Reduction in skyrmion density
MUA	Selective Adsorption	No change in skyrmion diffusion

S9 Video of skyrmion diffusion

Supplementary video 1 displays a video of typical skyrmion diffusion in our system. The sample in the video is of the stack: Ta(4)/Co20Fe60B20(0.9)/Ta(0.08)/MgO(1.5)/Ta(2)/Au(3nm).
